# Overview of Recombinant Tick Vaccines and Perspectives on the Use of Plant-Made Vaccines to Control Ticks of Veterinary Importance

**DOI:** 10.3390/vaccines12101178

**Published:** 2024-10-17

**Authors:** Edgar Trujillo, Abel Ramos-Vega, Elizabeth Monreal-Escalante, Consuelo Almazán, Carlos Angulo

**Affiliations:** 1 Immunology & Vaccinology Group, Centro de Investigaciones Biológicas del Noroeste, S.C. (CIBNOR), Instituto Politécnico Nacional 195, Playa Palo de Santa Rita Sur, La Paz 23096, BCS, Mexico; etrujillo@pg.cibnor.mx (E.T.); aramos@pg.cibnor.mx (A.R.-V.); emonreal@cibnor.mx (E.M.-E.); 2 Laboratorio Nacional CONAHCYT de Generación de Vacunas Veterinarias y Servicios de Diagnóstico (LNC-GVD), Centro de Investigaciones Biológicas del Noroeste, S.C., Instituto Politécnico Nacional 195, Playa Palo de Santa Rita Sur, La Paz 23096, BCS, Mexico; 3 Centro de Investigación en Ciencia Aplicada y Tecnología Avanzada (CICATA) Unidad Morelos del Instituto Politécnico Nacional (IPN), Boulevard de la Tecnología No.1036, Xochitepec 62790, MOR, Mexico; 4 CONAHCYT-Centro de Investigaciones Biológicas del Noroeste (CIBNOR), Av. Instituto Politécnico Nacional 195, Playa Palo de Santa Rita Sur, La Paz 23096, BCS, Mexico; 5 Immunology and Vaccines Laboratory, College of Natural Sciences, Autonomous University of Queretaro, Santiago de Queretaro 76230, QRO, Mexico

**Keywords:** veterinary diseases, immune system, innovative vaccine technology, oral delivery, animal health

## Abstract

Ticks are obligate hematophagous ectoparasites that affect animals, and some of them transmit a wide range of pathogens including viruses, bacteria, and protozoa to both animals and humans. Several vaccines have shown immunogenicity and protective efficacy against ticks in animal models and definitive hosts. After several decades on anti-tick vaccine research, only a commercial vaccine based on a recombinant antigen is currently available. In this context, plants offer three decades of research and development on recombinant vaccine production to immunize hosts and as a delivery vehicle platform. Despite the experimental advances in plant-made vaccines to control several parasitosis and infectious diseases, no vaccine prototype has been developed against ticks. This review examines a panorama of ticks of veterinary importance, recombinant vaccine experimental developments, plant-made vaccine platforms, and perspectives on using this technology as well as the opportunities and limitations in the field of tick vaccine research.

## 1. Introduction

Ticks are obligate hematophagous ectoparasites of terrestrial vertebrates that are involved in the transmission of a widely diverse array of pathogens including viruses, bacteria, fungi, and protozoans [1]. They are the most important disease vectors after mosquitoes [2]. Ticks included in the family Ixodidae are the most important vectors of diseases affecting domestic animals. From this, the genera *Rhipicephalus*, *Ixodes*, and *Haemaphysalis* are the most important in terms of economic losses and transmitted diseases to animals and humans.

The genus *Rhipicephalus* includes an important group of ticks of veterinary importance. For instance, the cattle tick *Rhipicephalus microplus* is one of the most important pests of livestock [3]. Direct effects of *R. microplus* include tick worry, blood consumption, damage to hides and udders, inoculation of toxins, and decreases in weight and milk production [4]. Indirect losses include transmission of diseases such as bovine anaplasmosis and babesiosis, the most devastating diseases of cattle raised in torrid zones [5]. Globally, economic losses due to *R. microplus* have been calculated at USD 13.9–18.7 billion per year [6].

The genus *Ixodes* includes the most important group of ticks of medical importance. In the American continent, *Ixodes scapularis* is the vector of Lyme disease, human babesiosis, and human anaplasmosis, considered the most prevalent tick-borne diseases in the United States and Canada. In addition, this tick is involved in the transmission of *Borrelia miyamotoi* disease, Powassan virus disease, and ehrlichiosis [7]. In Europe, *I. ricinus* is a vector of Lyme disease, tick-borne encephalitis, and human granulocytic anaplasmosis in humans, and tick-borne fever in sheep [8]. In Asia, *I. persulcatus* is the vector of Lyme disease, and tick-borne encephalitis and several pathogens from the genera *Borrelia*, *Rickettsia*, *Anaplasma*, *Coxiella*, and *Ehrlichia* [9].

The genus *Haemaphysalis* includes invasive ticks, such as the invasive tick *H. longicornis*, which is native to Asia and has been spread in Australia, other Pacific Islands, and 18 states, including the Northeast, Midwest, and Southeast regions of the United States. This tick feeds on a huge range of vertebrate hosts including humans and is the tick that transmits the most pathogens, including organisms from the genera *Babesia*, *Theileria*, *Anaplasma*, *Rickettsia*, and the virus Dabie bandavirus, the causative agent of severe fever with thrombocytopenia in cattle and humans (SFST) [10].

During the last century, the most common way to control ticks has been relayed in the application of acaricides. However, the intensive use of acaricides and poor management of these chemicals have contributed to the genetic selection of resistant tick populations, and in many cases, multiple resistance, directly impacting the environment by contamination of land and water, having negative effects on insects beneficial to wildlife, and introducing harmful residues in products and subproducts that are destined for human consumption [11]. Currently, resistance to all acaricide groups available in the market has been documented [12]. Therefore, anti-tick vaccines represent a feasible, affordable and environmentally friendly alternative for tick control to prevent tick infestations and tick-borne pathogens, and to mitigate acaricide resistance. Despite vaccines being a cost-effective control method and an ecologically friendly approach to prevent infestations and decrease parasite burden on cattle in infested areas, they are unavailable for all producers, or their distribution is limited to certain areas in Mexico [11].

The proof of concept that tick vaccines could be used to prevent tick infestations in animal hosts exposed to ticks was described by Allen and Humpreys [13] in guinea pigs and cattle that were immunized with crude extracts from gut and reproductive organs and then challenged with *Dermacentor andersoni* ticks, resulting in a decrease in tick weight, oviposition, and hatching. However, because *D. andersoni* is a three-host species, only adults were evaluated. Therefore, authors hypothesized that better results could be obtained using one-host ticks. This encouraged more studies, ending with the discovery of Bm86, a membrane-bound extracellular glycoprotein from the intestinal cells of the cattle tick, *Rhipicephalus microplus* [14]. The recombinant forms of Bm86 were included in formulations for the first vaccines against ticks, which were commercialized and distributed under the name TickGard and Gavac in Australia and Cuba, respectively [15].

Due to the fact that a variation in the efficacy of these vaccines was found in relation to geographical tick isolates, the identification of other tick vaccine candidates in *R. microplus* and other tick species continued, and other antigens such as Bm95 were identified [16]. Later, subolesin, an ortholog of the vertebrates’ akirines, was identified in *Ixodes scapularis* embryos [17]. This protein is conserved in several tick species and is involved in biological processes, including the immune response to infection by pathogens [18]. Immunization of several hosts with subolesin decreases the feeding and fertility of ticks fed on immunized animals [19]. Because subolesin is conserved among the various developmental tick stages of tick species, vaccination trials in several tick species have been performed, demonstrating the feasibility of tick control [20,21]. In a recent publication, an efficacy higher than 90% on *R. appendiculatus* in cattle vaccinated with subolesin was claimed [22].

According to the literature, most of the research on anti-tick vaccines has been performed on *R. microplus*, followed by *I. scapularis*, *Haemaphysalis longicornis*, *Amblyomma americanun*, and *I. ricinus* [23]. Despite vaccines being a cost-effective control method, and an ecologically friendly approach to prevent infestations and decrease parasite burden on cattle in naturally infested areas, even when several ticks and tick-borne pathogen vaccine candidates have been identified, the only vaccines currently available in the market are the Bm86-based Gavac^TM^ and Bovimune Ixovac^TM^. However, the distribution of these vaccines is limited to certain areas of a few Latin American countries [11].

The expression systems for recombinant Bm86-based vaccines are based on the use of *Escherichia coli* or the yeast *Pichia pastoris* platforms, respectively. Still, other methods such as plant expression systems have not been used. Plant-made vaccines are a technological platform with three decades of research and development that have allowed the delivery of several prominent recombinant vaccine candidates, including those against veterinary diseases, such as Rabies Virus [24], Avian influenza virus [25], Newcastle Disease [26], Foot-and-Mouth Disease Virus [27] and Enterotoxigenic *Escherichia coli* [28]. Plants can serve as vaccine production hosts and oral delivery vehicles. Currently, genetic tools are available for model and food-grade plants, offering attractive advantages to produce veterinary vaccines. Therefore, the expression of recombinant proteins for tick vaccines using plants is addressed herein and placed in perspective as another alternative to produce and make anti-tick vaccines based on recombinant proteins or peptides available for producers where access to any anti-tick vaccines is limited or nonexistent.

## 2. Recombinant Vaccines Against Ticks

Recombinant vaccines targeting different tick species have been included in the field of research and development for tick control over the past few decades. The primary aim of these studies is to identify and recombinantly express specific proteins that are crucial for the tick’s survival, feeding processes, decrease in reproduction, fertility and blocking the capacity to transmit diseases to hosts. These proteins, obtained from the salivary glands, digestive system, or reproductive organs, are critical for the tick’s capability to thrive and propagate. This section will delve into the numerous studies conducted on recombinant tick vaccines. The methodologies used, the experimental vaccine trials and the results observed in different hosts, including cattle, rabbits, and other animal models, will be briefly discussed.

### 2.1. Recombinant Vaccines Against Ixodes spp.

Labuda et al. [29] tested the 64TRP protein from *Rhipicephalus appendiculatus* as a vaccine against tick-borne encephalitis virus (TBEV) transmitted by *Ixodes ricinus*. The vaccine disrupted the tick’s feeding and killed engorged ticks by rupturing their midgut. Mice receiving 64TRP were protected from lethal TBEV infection, surpassing commercial vaccines in blocking transmission [29]. Synthetic peptide vaccines targeting neuropeptides like myoinhibitory peptide and SIFamide in *I. ricinus* induced IgG responses but showed no significant effects on nymphs in rodents, with limited data from sheep [30]. Almazán et al. [31] identified two vaccine candidates, IrLip1 and IrSPI, which generated strong antibody responses in mice and sheep but did not reduce tick infestations and unexpectedly increased tick engorgement. 

A Lyme disease vaccine was developed using a yeast-expressed library of *I. ricinus* salivary gland genes. Initial trials in livestock showed protection, but no similar effect was seen in rabbits [32]. Research on *Ixodes scapularis* used Salp14 as an antigen and found that mRNA lipid nanoparticles induced stronger immune responses compared to other vaccine methods [33]. The only vaccine against *Ixodes persulcatus*, targeting the CDK10 protein, reduced tick feeding and fertility in hamsters [34]. Salp25 is vital for *Borrelia burgdorferi* acquisition by *I. scapularis* ticks, detoxifying reactive oxygen species to aid B. burgdorferi survival at tick feeding sites [35]. Silencing salp25 or immunizing mice against it reduced Borrelia acquisition. Salp15 antiserum protects mice against B. burgdorferi, boosting antibody efficacy and enhancing phagocytic destruction of Salp15-coated Borrelia [36]. Another study identified a tick histamine release factor (tHRF), upregulated in infected ticks, that aids feeding. Silencing tHRF reduced tick feeding and Borrelia load, while immunizing mice against tHRF impaired tick feeding and pathogen transmission, enhancing vaccine potential [37]. Finally, a tick receptor produced in the gut of Ixodes called TROSPA was expressed in *Nicotiana benthamiana* and *E. coli* [38]. This example will be discussed further below (Table 1).

**Table 1:** Prototypes of recombinant vaccines against *Ixodes* spp. infection.

Arthropod Specie	Antigen Vaccine Prototype/ Expressión Platform	Pathogen	Animal Model and Immunization Schedule	Main Findings	Reference
*Ixodes ricinus* *Rhipicephalus appendiculatus*	Tick saliva protein (64TRPs) from *Rhipicephalus appendiculatus* expressed in *Escherichia coli*	Tick-borne encephalitis virus (TBEV)	Mice: One subcutaneous dose of 10 μg of 64TRP (alone or in cocktail with soluble, denatured protein or with C-terminal truncation), using Titermaxgold as adjuvant.	-Elicited a specific humoral response. -Protection against a lethal challenge with infected ticks. -Induced inflammatory immune responses in the tick feeding area.	[29]
*Ixodes scapularis*	Tick salivary protein—Salp15; expressed in *Escherichia coli*	*Borrelia burgdorferi*	Mice: A 10 μg subcutaneous dose of purified recombinant Salp15 emulsified in complete Freund’s adjuvant, boosted with 5 μg in incomplete Freund’s adjuvant, 2 weeks apart.	-Elicited a specific IgG humoral response. -Significant reduction in infection from tick-borne *Borrelia*.	[36]
*Ixodes scapularis*	25 kDa salivary gland protein (Salp25D) expressed in *Escherichia coli*	*Borrelia burgdorferi*	Mice: 10 μg of rSalp25D in complete Freund’s adjuvant and boosted twice at 2-week intervals with 10 μg of rSalp25D in incomplete Freund’s adjuvant.	-Reduced spirochete acquisition by ticks to threefold in comparison to the nonimmunized controls	[35]
*Ixodes scapularis*	Tick Saliva protein histamine release factor (tHRF) expressed in *Drosophila melanogaster*	*Borrelia burgdorferi*	Mice: One subcutaneous dose of 10 μg of purified recombinant tHRF suspended in complete Freund’s adjuvant, boosted with 5 μg of antigen suspended in incomplete Freund’s adjuvant every two weeks.	-Elicited a specific IgG humoral response. -Tick weights decreased, spirochete load reduced, and 20–33% of immunized mice were PCR-negative.	[37]
*Ixodes scapularis* *I. ricinus*	Gut protein TROSPA expressed in *Escherichia coli* and *Nicotiana benthamiana*	*Borrelia burgdorferi*	Rats: three oral immunizations with 200 μg of the purified TROSPA in PBS buffer with or without 1 unit of GEM particles as adjuvant (Gram-positive enhancer matrix, from *Lactococcus lactis*), at 14-day intervals	-Elicited a specific IgG humoral response. -TROSPA protein was not detected in the plant leaves.	[38]
*Ixodes persulcatus*	The Cyclin-dependent kinases IpCDK10 expressed in *Escherichia coli*		Syrian hamsters: three subcutaneous doses of 100 μg of recombinant IpCDK10, using Freund’s complete adjuvant for priming, and Freund’s incomplete adjuvant for the two boosts, at 14-day intervals.	Ticks fed the IpCDK10 vaccine group were significantly smaller in size and weighed less, as well as a 50% decrease in egg weight, and tick egg hatching was 80% lower.	[34]
*Ixodes ricinus*	MAPs SIFa and MIP neuropeptides fused to the PADRE peptide	*Anaplasma phagocytophilum*	Mice: three subcutaneous doses with 10 µg of MAPs, with 14-day intervals. Sheep: three intramuscular doses 50 µg of MAPs, with 15-day intervals, using Montanide™ ISA 201 VG as adjuvant.	-Elicited a specific IgG humoral response. -There were no changes in nymphs fed by mice or sheep, or in bacterial transmission.	[30]
*Ixodes ricinus*	The salivary proteins serine protease inhibitor (IrSPI) and lipocalin 1 (IrLip1), expressed in *Drosophila* S2 cells	*Anaplasma phagocytophilum*	Mice: three subcutaneous doses with 10 µg of MAPs, with 14-day intervals. Sheep: three intramuscular doses 50 µg of MAPs, with 15-day intervals. Both using Montanide™ ISA 201 VG as adjuvant.	-Elicited a specific IgG humoral response. -No protection against infestation by *I. ricinus* nymphs and larva in mice and sheep was observed, enhancing tick engorgement and molting and decreasing tick mortality.	[31]
*Ixodes scapularis*	The salivary protein 14 (Salp14) using mRNA lipid nanoparticles (LNPs), plasmid DNA, or recombinant protein (expressed in *Drosophila*).	N/A	Guinea pigs: Three intradermal doses of 20 μg of Salp14 mRNA-LNPs, 80 μg of plasmid DNA encoding Salp14 or empty plasmid constructions VR2010, 20 μg of recombinant Salp14 or Ovalbumin (OVA control), and sustained immunization with 20 μg of recombinant Salp14 over the course of one week.	-Elicited a specific IgG humoral response. -mRNA-LNP vaccination elicited erythema at the tick bite site, more pronounced than DNA or protein immunizations.	[33]
*Ixodes ricinus*	The salivary proteins V5H126, B7PDE7, A0A0K8R6W3, expressed in *Escherichia coli*	N/A	Cows: two doses with 100 µg of each antigen separately (V5H126, B7PDE7, A0A0K8R6W3) or, in a second experiment, with the three antigens combined, at interval of six weeks, using saponin in 1 mL Montanide ISA V50 as adjuvants. Rabbits: three doses 50 µg of V5H126, B7PDE7 and A0A0K8R6W3, at three weeks intervals, using incomplete Freund’s for priming and incomplete Freund’s adjuvant for boosters.	-Elicited a specific IgG humoral response. -No signifcant reduction in tick parameters was observed afer single-antigen immunization, but vaccination with all three antigens resulted in a signifcant reduction in the number of engorged adult ticks as well as their engorgement weights.	[32]

N/A: No Apply.

### 2.2. Recombinant Vaccines Against Rhipicephalus spp.

*Rhipicephalus microplus* is the major cattle tick responsible for significant economic losses in the cattle industry. The efficacy of various recombinant antigenic proteins and their potential in reducing tick infestations and controlling the pathogens transmitted by ticks are presented. One of the most widely studied vaccines is based on the Bm86 glycoprotein, which is located on the surface of *R. microplus* intestinal cells [39]. Bm86 is encoded by a 1982 bp gene, which produces a 650-amino acid protein, including a 19-amino acid signal peptide and a 23-amino acid hydrophobic segment near the carboxylic end [14]. Bm86, when expressed in *Escherichia coli* and administered as a vaccine, has demonstrated efficacy rates of 70–90% against tick infestations [40]. Similarly, Bm86 produced in *Pichia pastoris* achieved comparable results in controlling tick populations [41]. However, its effectiveness in South America was lower, prompting the development of Bm95, a Bm86 homolog that achieved 58–89% efficacy [16]. Additionally, Bm86 orthologues, such as Ba86, have shown up to 90% similarity and cross-reactivity with Bm86, resulting in higher efficacy against *Rhipicephalus annulatus* compared to *R. microplus* [42,43] (Table 2).

Beyond Bm86, various other antigens have been evaluated for their immunoprotective potential. The proteins TROSPA, SILK, and Q38 were tested against *R. microplus* infestations, with Q38 showing a 75% reduction in tick infestations, and SILK reducing oviposition by 62% [44]. Subolesin, a highly conserved protein among tick species, is an orthologue of insect akirin and is present among the different developmental stages as well as among tick species [45]. It was identified using a cDNA library from *I. scapularis* embryonic cells [17]. Functional studies determined that Subolesin decreased tick feeding and fertility and showed a 60% reduction in tick infestations and 51–60% protective efficacy against *R. microplus* and *R. annulatus* [20]. To improve efficacy, a subolesin peptide was designed and tested in vaccine preparations under field conditions and tested in field trials, resulting in 67% efficacy against *R. microplus* [21].

Other antigenic proteins have demonstrated promising results in cattle vaccination trials. For example, the metzincin protein BrRm-MP4, when used as a vaccine, impaired tick feeding and reduced reproductive traits, offering 60% protection against tick infestations [46]. Similarly, sialoproteins, which facilitate parasitism and evade the host immune system, were tested in alum-adjuvanted vaccines, achieving 73.2% efficacy [47]. Another example is the voltage-dependent anion-selective channel (VDAC) identified in the midgut of *R. microplus*, a mitochondrial membrane protein of 30–32 kDa present in eukaryotes [48]. Vaccination with recombinant voltage-dependent anion-selective channels (VDACs) resulted in 82% efficacy, primarily by reducing egg fertility [49].

Innovative vaccine development has also explored new targets, such as Cys-loop receptors, glycine-like receptors, and glutamate receptors, which showed moderate efficacy (25–33%) in controlling *R. microplus* infestations [50]. Additionally, a peptide derived from Serpin RmS-17 exhibited 79% efficacy in reducing tick numbers and oviposition, surpassing the 62% efficacy of the Bm86 vaccine [51].

Efforts have also been made to develop vaccines offering protection against multiple tick species. Calreticulin (CRT) proteins from *R. microplus* and *Haemaphysalis longicornis* demonstrated cross-reactivity, with antibodies from vaccinated cattle and mice recognizing both CRT proteins [52]. Another multi-antigen vaccine, combining vitellin-degrading cysteine endopeptidase (VTDCE), yolk pro-cathepsin (BYC), and glutathione S-transferase (GST-Hl), provided greater protection against *R. microplus* than individual antigen vaccines [53]. Cross-protective potential has also been evaluated using BM86 from *R. microplus* along with subolesin and tropomyosin from *Hyalomma anatolicum*, achieving high efficacy against both tick species [54].

Additional promising vaccine candidates include MSP1a chimeric proteins, which achieved 64–81% protection against *R. microplus* infestations [55], ferritin (FER2), which is crucial in iron metabolism and provided 64–72% protection [56,57], and recombinant aquaporins, with 68–75% efficacy in Brazilian trials [58]. Chitinase peptides, targeting the molting process of ticks, were also evaluated, achieving 71% protection in cattle [59].

Recombinant vaccines against *Rhipicephalus sanguineus* and *Rhipicephalus appendiculatus* have also been explored. For instance, a 20-amino acid peptide from P0, a ribosomal protein essential for tick viability, achieved 90% efficacy in rabbits and 85% efficacy in dogs [60,61]. Similarly, Bm86 was tested in dogs, leading to significant reductions in larval, nymphal, and adult tick collection rates [62]. In the case of *R. appendiculatus*, cystatin proteins demonstrated their potential as vaccine candidates by inhibiting cathepsins involved in tick digestion and blood processing, reducing engorged adult females by 11.5% [63].

**Table 2:** Prototypes of recombinant vaccines against *Rhipicephalus* spp. infection.

Arthropod Specie	Antigen Vaccine Prototype/ Expressión Platform	Pathogen	Animal Model and Immunization Schedule	Main Findings	Reference
*Rhipicephalus microplus* and *Haemaphysalis longicornis*	Saliva protein Calreticulin; expressed in *Escherichia coli*	N/A	Mice: three intraperitoneal doses of 100 μg of protein emulsified in Freund’s incomplete adjuvant, at 14-day intervals. Bovines: three subcutaneous doses of 100 μg plus 1 mL of oil adjuvant (Montanide 888 and Marcol 52), at 14-day intervals. Doses were administered 14 days apart.	-Elicited a specific humoral response.	[52]
*Rhipicephalus sanguineus*	Peptide of an immunogenic region of the ribosomal protein P0	N/A	Rabbits: four subcutaneous doses of pP0- KLH conjugate at doses of 500 μg/animal (equivalent to 250 μg pP0/animal) emulsified with VG Montanide 888 adjuvant (60/40 proportion of immunogen/adjuvant), on days 0, 21, 36 and 60.	-Elicited a specific IgG humoral response. -Decrease in the viability of recently molted nymphs of larvae fed with vaccinated rabbits, and a significant reduction in the number of adults and eggs that hatched, showing an overall efficacy of 90%.	[60]
*Rhipicephalus sanguineus.*	The midgut protein Bm86, expressed in *Pichia* *pastoris*	N/A	Dogs: two intramuscular doses of 50 μg of recombinant Bm86, with 21-day interval.	-Elicited a specific IgG humoral response. -Collection rates of larvae, nymphs and adult females fed with vaccinated dogs were significantly reduced (*p* < 0.05) by 38%, 29% and 31%, respectively, as well as in the weight of engorged females and in mass of eggs, in the conversion efficiency rate to eggs, but not in the hatching rate of ticks fed with immunized dogs.	[62]
*Rhipicephalus microplus*	The egg-associated proteins VTDCE and BYC from *R. microplus*, and GST-Hl from *Haemaphysalis longicornis*, expressed in *Escherichia coli.*	N/A.	Cattle: Four subcutaneous doses of 200 μg each antigen, emulsified with 0.5 mL of the adjuvant Montanide 888 and Marcol 52, with 21-day intervals.	-Elicited a specific IgG humoral response. -Vaccinated cattle show greater weight gain, as well as a significant reduction in the number of semi-engorged ticks.	[53]
*Rhipicephalus microplus*	TROSPA, salivary protein SILK, SUB and Q38 chimera from *R. microplus*, expressed in *Escherichia coli*	*Babesia bigemina*	Cattle: 3 doses (days 0, 28 and 49) containing 100 ug of purified recombinant proteins with the Montanide ISA 50 V as adjuvant.	-Elicited a specific IgG humoral response. -Reduction in tick infestations and oviposition with vaccine efficacies of 75% (Q38), 62% (SILK) and 60% (SUB), Q38, TROSPA and SUB reduced *B. bigemina* DNA levels in ticks, while vaccination with SILK and SUB resulted in lower levels of *A. marginale* DNA.	[44]
*Rhipicephalus microplus*	The aquaporin protein RmAQP1, expressed in *Pichia pastoris*		Cattle: three intramuscular doses of 100 μg of the recombinant protein using with Montanide ISA 61 VG as adjuvant, with two-week intervals.	-Elicited a specific IgG humoral response. -Vaccine demonstrated 75% and 68% of efficacy in reducing the numbers of adult female ticks.	[58]
*Rhipicephalus microplus*	The Metalloprotease BrRm-MP4 expressed in *Escherichia coli*		Calves: Two subcutaneous doses of 100 μg of purified rBrRm-MP4, and two more doses with 200 μg of rBrRm-MP4, using Montanide 888 as adjuvants, at 15-day intervals.	-Elicited a specific IgG humoral response. -Vaccination significantly decreased the number of engorged females and their reproductive potential, representing 60% overall protection.	[46]
*Rhipicephalus microplus*	The salivary proteins Rm39, Rm180, Rm239, and Rm76 expressed in *Escherichia coli*		Calves: Antigens Rm39, Rm180, and Rm239 were prepared separately in a mixture containing 100 μg of recombinant protein, while Rm76 was prepared as a 25 µg dose. Three intramuscular doses to the neck with the four recombinant proteins (in separate injections) at 3-week intervals (days 0, 21 and 42 of the trial), using aluminium hydroxide as adjuvant.	-Elicited a specific IgG humoral response. -Significant reduction in the number of female ticks (52.5%) and tick engorgement weight (55.2%) in vaccinated calves, demonstrating an overall protection of 73.2%.	[47]
*Rhipicephalus appendiculatus*	The cystatin Racys2a, expressed in *Escherichia coli*	N/A	Rabbits: three subcutaneous doses of 200 μg of recombinant protein at two-week intervals, using Marcol/Montanide as adjuvant.	-Elicited a specific IgG humoral response. -Vaccination caused damage to the gut, salivary gland and ovary tissues in ticks, reducing the number of fully engorged adult females in 11.5%.	[63]
*Rhipicephalus microplus*	Cys-loop receptors: N-terminal domains of a glutamate receptor and of a glycine-like receptor, expressed in *Escherichia coli*	N/A	Mice: Four doses of 20 μg of recombinant N-terminal ECD of the glutamate-activated receptor (rGluCl, 4 mice) or recombinant N-terminal ECD of the glycine-like receptor (rGlyR, 3 mice), the first dose with complete Freund’s adjuvant, and the subsequent doses with incomplete Freund’s adjuvant, at two-week intervals. Cattle: Three intramuscular doses (days 1, 30, and 50) of 100 µg/dose of rGluCl or rGlyR proteins, using Montanide ISA 50 V2 as adjuvant.	-Elicited a moderate humoral IgG response on vaccinated cattle. -Vaccine efficacies of 33% and 25% were obtained for the glutamate receptor and the glycine-like receptor, respectively.	[50]
*Rhipicephalus microplus*	The salivary protein Serpin RmS-17, expressed in *Escherichia coli*	N/A	Rabbits: Three subcutaneous doses of RmS-17 peptide, *R. microplus* recombinant antigen Bm86.	-Elicited a specific humoral IgG response. -Vaccine efficacy of 79% by the reductions in adult tick number, oviposition, and egg fertility.	[51]
*Rhipicephalus microplus and Hyalomma anatolicum*	BM86, Subolesin and tropomyosin, expressed in *Kluyveromyces lactis*	N/A	Calves: Three intramuscular doses of 100 µg each protein using Montanide ISA 50V2 as adjuvant, at thirty-day intervals.	-Elicited a specific humoral IgG response. -Vaccine efficacy was 87.2% and 86.2% against *H. anatolicum* larvae and adults, respectively, and 86.7% against *R. microplus*.	[54]
*Rhipicephalus microplus*	Mitochondrial protein VDAC expressed in *Escherichia coli*	*Babesia bigemina*	Cattle: 3 subcutaneous doses of rBmVDAC 100 µg/dose, with 21-day intervals, using Montanide ISA 71VG as an adjuvant.	-Elicited a specific humoral IgG response. −82% efficacy against *R. microplus.*	[49]
*Rhipicephalus microplus* and *Rhipicephalus sanguineus* s.l.	A synthetic 20 amino of the acid peptide acidic ribosomal protein P0 of *Rhipicephalus* spp., conjugated to Bm86 expressed in *P. pastoris*	N/A	Dogs: Three subcutaneous immunizations (on days 0, 21 and 36) with 500 μg of pP0–Bm86 conjugate, using Montanide ISA 50 as an adjuvant.	-Elicited a specific humoral IgG response. -Efficacies of around 90% against *Rhipicephalus microplus* and *Rhipicephalus sanguineus* s.l.	[61]
*Rhipicephalus microplus*	Polypeptide Bm86 expressed in *Escherichia coli*	N/A	Cattle: Three subcutaneous doses with 100 μg (on days 0, 30 and 49), using Montanide ISA 50 V as an adjuvant.	-Elicited a specific humoral IgG response. -Vaccine efficacy of 58%.	[40]

N/A: No Apply.

While Bm86 remains a cornerstone of anti-tick vaccine development, other proteins and multi-antigen formulations have demonstrated considerable promise. These novel approaches aim to enhance protection against a broader range of tick species, offering hope for more effective control of tick-borne diseases and infestations.

### 2.3. Recombinant Vaccines Against Haemaphysalis spp.

The acid phosphatase HL-3 from *Haemaphysalis longicornis* was evaluated as a vaccine candidate. Recombinant HL-3 (rHL-3), expressed in *E. coli*, was used to immunize rabbits, leading to a 28% tick mortality rate and a 10.6% reduction in adult tick weight, suggesting its role in immunity and potential as a vaccine antigen [64]. The serine protease Longistatin, another salivary gland protein from *H. longicornis*, showed 73% effectiveness in reducing tick infestation in vaccinated mice, lowering tick repletion, body weight, and nymph molting [65].

Additionally, rabbits immunized with paramyosin (rPmy) and a peptide (KLH-LEE) showed reductions in tick engorgement weight (8.87%), oviposition (26.83%), and hatchability (38.86%), with vaccine efficacies of 60.37% and 70.86%, respectively [66]. Cross-reactivity studies found that a lipocalin homologue (HlLIP) from *H. longicornis* shared high sequence homology with *Ixodes persulcatus*, reducing tick engorged weight and reproductive parameters in rabbits, with a 60.17% efficacy [67]. Lastly, recombinant triosephosphate isomerase (rHlTIM) from *H. longicornis* reduced tick weight, oviposition, and egg hatching by 8.6%, 35.4%, and 17.3%, respectively, with an overall efficacy of 50.9% [68] (Table 3).

### 2.4. Recombinant Vaccines Against Amblyomma spp.

A study using RNA interference (RNAi) on a *Amblyomma americanum* cDNA library identified four tick-protective antigens: threonyl-tRNA synthetase, 60S ribosomal proteins L13a and L13e, and interphase cytoplasm foci protein 45. Cattle vaccinated with these recombinant proteins and subolesin showed over 30% efficacy, with recombinant 2G7 or subolesin achieving more than 55% control against adult ticks [69].

In *Amblyomma sculptum*, three salivary proteins (AsKunitz, As8.9kDa, and AsBasicTail) were characterized and assessed as vaccine candidates, demonstrating significant inhibition of key enzymes and protection against infestation in mice, with efficacy rates between 59.4% and 85% and nymph mortality reaching 70–100% [70] (Table 4).

### 2.5. Recombinant Vaccines Against Hyalomma spp.

Cathepsin L-like cysteine protease (CPL) was assessed for its potential use in a bivalent vaccine targeting *Hyalomma anatolicum* and *H. asiaticum*. This protein is a key hemoglobinase involved in blood digestion from hosts. CPL from *H. anatolicum* (HanCPL) showed over 90% similarity to *H. asiaticum* CPL (HasCPL). In vitro experiments demonstrated that anti-HasCPL sera cross-reacted with native proteins across different developmental stages and tissues of both *H. asiaticum* and *H. anatolicum*. Additionally, rabbits immunized with recombinant HasCPL (rHasCPL) showed partial cross-protection (54.8%) against *H. anatolicum* infestation [71] (Table 5).

The recombinant tick vaccines described demonstrate promising efficacy in controlling tick infestations by targeting various tick antigens, including Bm86, subolesin, and salivary proteins. These vaccines showed significant reductions in tick infestations, oviposition, and reproductive success, with efficacies ranging from 30% to over 85%. This highlights the potential of recombinant proteins in developing effective anti-tick vaccines for both livestock and companion animals, particularly in Latin American countries (Table 6).

## 3. Plant as Recombinant Vaccine Production Host and Delivery

Plants have emerged as promising hosts for producing and delivering recombinant vaccines. This approach, known as plant molecular pharming, involves using plant cells or whole plants as platforms to produce vaccines and other therapeutic proteins [72]. This platform has several advantages, including lower production costs, scalability, and reduced risk of contamination with pathogens like prions and endotoxins, which are concerns in mammalian and bacterial systems.

Some advantages described, like bioencapsulation, could favor antigen uptake and display resistance to degradation, or plant metabolites could exert adjuvant activity. Plant compounds, such as polysaccharides, can exert mucoadhesive properties, and differential glycosylation conferred by the plant cell machinery could enhance immunogenicity [73].

The general methodological strategy comprises elements and approaches selected to reach the desirable objective. Firstly, a gene-coding antigen with demonstrated protective efficacy can be identified by scientific reports, or a chimera can be designed based on several antigens and epitopes. Commonly, the gene is codon-optimized and then synthesized considering appropriate restriction sites for cloning procedures. Additional genetic elements can be included in up- and downstream genes, such as a sequence to drive the mRNA to ribosomes for translation, a retention signal if the protein must be retained in the cell, a tag for purification if this is the case, and a sequence-coding adjuvant to potentiate the immune response, among others [74]. Moreover, it can improve the expression according to the cellular destination where the antigen is processed, for example, chloroplast, endoplasmic reticulum/secretion [75], and a significant portion of recombinant vaccine production achieved through tobacco chloroplast transformation relies on this method, such as cholera toxin B subunit [76] or human papillomavirus L1 virus-like particle (VLP) [77]. Some vaccine designs include the fusion of the antigen of interest with other proteins that considerably improve its expression in plants and stability [78].

The expression vector should be selected considering whether the approach is nuclear or plastid genetic transformation. However, it is possible to express recombinant antigens in plants in a transient form or cell suspension instead of generating stable transgenic plants [78,79]. This option could be appropriate to avoid the neutralization and degradation of the transgenic plant, which can occur as a rejection of exogenous antigen. Moreover, the decision will also condition the method to transfer the genetic construction comprising the vector and the gene, for which *Agrobacterium tumefaciens*- and biobalistic-mediated methods are the most used. Typically, model plants, such as tobacco and Arabidopsis, are employed to explore the potential of a given antigen due to their high transformation efficiency and regeneration. Plant-made antigens are detected by Western blot and quantified by ELISA. Then, the proof of concept is followed up with food-grade plants including rice, maize, and alfalfa, among many others [74].

In this context, plant-based vaccines are especially suitable for oral immunization, providing the advantage of convenient, needleless administration and reducing the costs needed for purification of the antigens, and these vaccines are easily freeze-dried for long-term storage at room temperature [80]. It has been proposed that plant-produced vaccines are protected from the acid environment of the stomach by the plant cell wall and are then slowly released in the gut [81], inducing host mucosal and systemic immunity after uptake by M cells in the follicle-associated epithelium [82]. Immune tolerance is considered a potential barrier to the development of edible vaccines because of the high-dosage oral administration that is usually required. Therefore, the optimum antigen dosage needs to be carefully determined [83].

Despite efforts in plant-made vaccine development, there are still challenges that hinder the realization of manufacturing-approved and safe products, among them relatively low yields and achieving elicitation of a robust response toward the immunogen [84].

### Examples of Plant-Made Vaccines Against Arthropods

Most potential recombinant tick vaccines have been produced using conventional expression systems, including bacteria (*E. coli*), yeast (*Pichia pastoris* and *Kluyveromyces lactis*), and insect cells (Drosophila S2 cells). In plants, to our knowledge, the first attempt was reported in a study that described the gene expression of an antigen with vaccine potential against ticks. TROSPA (a tick receptor produced in the intestine of *Ixodes* ticks that binds to Outer surface protein A (OspA)) expressed by *B. burgdorferi* allowed colonization and survival of this spirochete in ticks; thus, it was considered a potential vaccine candidate against Lyme disease. Experiments to express TROSPA in the plant *Nicotiana benthamiana* and *E. coli* were performed by Figlerowicz et al. [38]. Unfortunately, TROSPA protein was not detected in *N. benthamiana* leaves, but in *E. coli*, demonstrating the ability to bind to OspA from several *Borrelia* species and immunogenicity in rats. The N-terminal part of TROSPA does not participate in OspA interaction, and reducing TROSPA’s negative charge impairs this binding. Recombinant TROSPA effectively forms complexes with OspA and generates specific IgG in orally vaccinated rats. The production of TROSPA was efficient in *E. coli* cells; although correctly spliced mRNA was confirmed in the plant cells, the total protein isolated from these cells did not show its presence. The authors suggested that the non-detection of plant-produced TROSPA could be due to the use of sera from animals injected with bacterial recombinant protein, antibodies targeting non-glycosylated protein, ineffective production of TROSPA in the plant system, or rapid degradation by plant proteases [38].

To improve the expression of TROSPA and other antigens in plants, optimizing codon usage specific to the plant expression system could enhance protein yield [85]. Using stronger plant promoters, such as CaMV 35S or tissue-specific promoters, may also boost expression [86]. Co-expression with chaperones or fusion to stabilizing tags could improve protein folding and stability [87]. Additionally, glycosylation patterns should be considered for proper antigenicity. Given the promising results from other recombinant antigens (e.g., Bm86, subolesin), further studies should explore the plant-based expression of these proven candidates to enhance immune response and protection against ticks.

To date, only one attempt of a plant-produced vaccine against ticks has been reported; this platform has been extensively explored for producing vaccines against pathogens transmitted by arthropods, primarily those transmitted by mosquitoes. Most of the research developed in this area includes the development of vaccines against parasitic diseases such as malaria [88,89,90,91,92,93] and leishmaniasis [94,95], as well as those associated with the *Flavivirus* genus such as dengue [96,97,98,99], Zika [100,101,102], yellow fever [103], West Nile virus [104,105,106,107], and Japanese encephalitis [108]. Additionally, research on plant-made vaccines has been reported for diseases caused by alphaviruses like Chikungunya [109,110], and other diseases such as bubonic plague, spread by fleas [111,112,113].

## 4. Opportunities and Limitations in the Field for Plant-Made Vaccines Against Ticks

**Figure 1:**
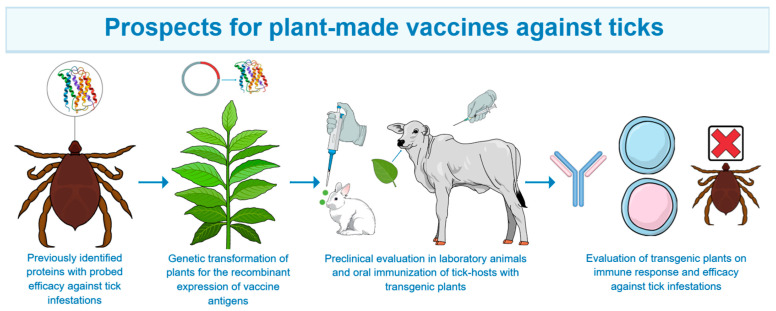
Schematic representation of step-by-step approach for plant-made vaccine development against ticks of veterinary importance.

Plant-made vaccines are an alternative that has not been explored to control ticks of veterinary importance (Figure 1). Considering that several antigens have been identified, that evidence of the efficacy against several tick species is available, that anti-tick vaccines are still not easily affordable for producers, and the existent experience gained on plant-made vaccines against several vector-borne pathogens, in this review, a perspective of the use of plant-made vaccines is addressed. Adapting this methodology opens a field of opportunities in tick vaccine development, the results of which may be used to decrease tick infestations in regions where vaccines are unavailable, and tick control relies only on acaricides. The pioneering study of Figlerowicz et al. [38] has demonstrated that challenges must be overcome to successfully produce antigens from ticks. However, over 35 years of plant-made vaccines against animal and human diseases shed light on the application of this technology. Many plant-made vaccines have entered clinical trials, and some have reached government licensing, such as COVIFENZ^®^ of Medicago company to prevent COVID-19 [114]. Thus, plant-made vaccines have reached clinical trials and been commercialized for human use [115,116], making this technology a reality for the benefit of humankind.

Advantages from plants can be exploited as a vaccine biofactory since these organisms are a natural source for industrial purposes. Their molecular mechanisms let them synthesize functional compounds, such as recombinant vaccines. Additionally, some plants produce immunostimulatory compounds that enhance immune responses and serve as adjuvants of vaccines. The major challenge of plant-made vaccines is to achieve high recombinant protein yields. In this arena, the availability of genetic engineering tools, products, and protocols, especially those related to model plants like tobacco, is an advantage. However, limitations in genetic manipulation exist for many less-studied plants. This situation represents an obstacle to transformation efficiency and developing species-specific protocols for transgenesis becomes a big hurdle. Nonetheless, plant-made vaccine technology is permanently advanced in scientific innovation and knowledge. For instance, strong inductive and constitutive promoters, terminators, retention signals, transformation techniques, and glycoengineering tools, among others, have been developed and continuously innovated [117,118]. Codon optimization of gene sequences has been recommended to improve recombinant vaccine production, but Maclean et al. [119] demonstrated that the opposite could be true, and this should be considered.

Regulations for plant-made vaccines against veterinary diseases are less restrictive than those for human diseases, which is an advantage for industrial adoption. In line with this, Good Manufacturing Practices (GMP) should comply with regulations for optimal plant-made anti-tick vaccine production [120]. Therefore, genetic transformation, growing, and harvest conditions should be optimized to obtain the highest yields, quality, and cost-effective vaccines. Fortunately, the knowledge behind agriculture for most of the used plants to produce recombinant vaccines brings a huge advance, leading to a focus on optimizing genetic transformation. A concern is related to the biosecurity of transgenic plants [121]. This issue must be solved through containment systems, control protocols, and strict monitoring to avoid any risk of an accidental escape that could affect the environment [122]. If the vaccine must be purified for parenteral application, this issue will represent additional GMP labor. Remarkably, oral vaccination is an advantage because plant-made vaccines can be directly administered, without the need for purification procedures, therefore decreasing the costs of production, while other costs are avoided, such as the need for trained personnel, veterinary materials, etc. Additionally, encapsulated antigens in the plant cells are generally thermostable for oral delivery and can avoid the cold chain to maintain vaccine functionality. This is a big advantage that would greatly impact veterinary vaccines, especially when vaccine efficacy depends on the cold chain, which is difficult to maintain in tropical regions with extensive production systems, where tick infestations are high, and logistics and cost for cold vaccine distribution may be significantly reduced.

A key challenge of plant-made oral vaccines is immunotolerance due to the low immunogenicity of subunit vaccines. This phenomenon is due to the delicate balance between the food digestion activity, antigen immune recognition process, and the induction of immune responses at the gastrointestinal level. The main strategy to solve this problem has been through strong adjuvants, which can be part of the gene chimera or incorporated into the vaccine formulation [123]. An additional strategy to fence immunotolerance has been immunization schemes comprising oral (prime) and parenteral (boost) plant-made vaccine administration. This approach can stimulate mucosal and systemic immune responses, and it is especially desirable for mucosal pathogens.

### 4.1. Vaccines Against Tick-Borne Diseases and Plant-Made Vaccine Perspectives

**Figure 2:**
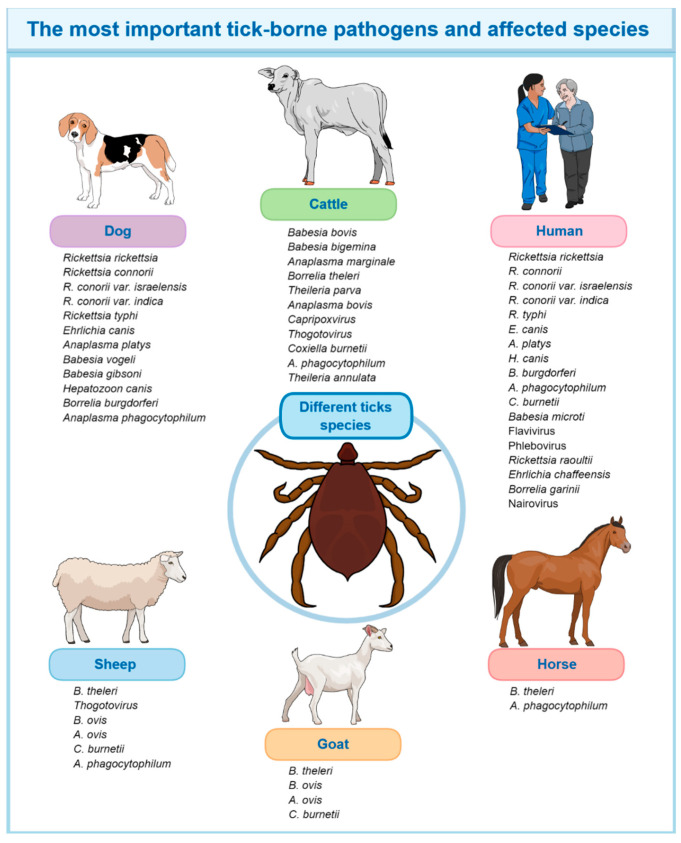
Tick-borne pathogens affect animals and humans.

In general, vaccination against ticks can also reduce the prevalence of tick-borne diseases (Figure 2), which is valid for any vaccine produced in available platforms, including plants. In this regard, Salp25 is crucial for *Borrelia burgdorferi* acquisition by *I. scapularis* ticks. Silencing salp25 in tick salivary glands or immunizing mice against Salp25 reduced *Borrelia* acquisition. Salp25 detoxifies reactive oxygen species, aiding *B. burgdorferi* survival at the tick feeding site. This shows that pathogens use arthropod molecules to overcome mammalian defenses and enter the vector [35]. Moreover, a follow-up study demonstrated that Salp15 antiserum provided protection to a mouse model against *B. burgdorferi* infectivity and enhanced the efficacy of antibodies targeting *B. burgdorferi* antigens. Additionally, Salp15 antiserum augmented the destruction of Salp15-coated *B. burgdorferi* by phagocytic cells, and immunized rodents were protected against tick-transmitted Borrelia. [36]. Another study carried out by this research group described a tick histamine release factor (tHRF) from *I. scapularis* and explored it as a vaccine. This protein, upregulated in *B. burgdorferi*-infected ticks, is detected in saliva and coincides with the fast-eating stage. Silencing tHRF diminished tick eating and reduced *B. burgdorferi* load in rodents. Mice immunized with tHRF or treated with tHRF antiserum showed decreased tick feeding efficiency and *B. burgdorferi* load. The tHRF binds to host basophils, stimulating histamine liberation, and potentially raising the blood stream to the tick bite point. Blocking tHRF could enhance vaccines against tick alimentation and pathogen spread [37]. In *A. marginale*, vaccination with Q38, TROSPA, and Subolesin diminished *B. bigemina* DNA quantities in ticks, while SILK and Subolesin reduced *A. marginale* DNA concentrations [44]. Therefore, an integrative strategy comprising antigens to fight against ticks and tick-borne diseases is ideal for dealing with diseases affecting food-producing animals.

### 4.2. Experience with Plant-Made Vaccines Against Parasites and Prospects Against Ticks

Vaccination in animal models and target hosts with several antigens has demonstrated low, moderate, and high protective efficacy, ranging from 25 to 90%. Those antigens showing the highest protective efficacy are candidates to be produced in plants for oral vaccination. The experience of plant-made vaccines developed against vector-borne human diseases must be considered to fight ticks of veterinary importance. For instance, several plants have expressed antigenic protein from *Plasmodium falciparum*, the causal agent of malaria transmitted by mosquitos. Successful plant expression of the sexual and asexual blood stage antigens (i.e., Pfs25, Pfs230, AMA1, MSP1) has been reported, demonstrating immunogenicity in animal models [38,88,89,92,93,124]. Furthermore, Pfs25 produced in tobacco plants and formulated with Alhydrogel^®^ elicited immunoglobulins that provided a complete transmission-blocking effect during a six-month study in mice [125]. Additionally, a Phase 1 study evaluated the safety, reactogenicity, and immunogenicity of the plant-made Pfs25 VLP vaccine with Alhydrogel^®^ adjuvant in healthy volunteers. No adverse effects like dose-related toxicity were seen after the vaccination with the plant-made Pfs25 VLP vaccine. Regarding immunogenicity, Pfs25 induced specific IgGs, but the antibodies had weak transmission-reducing activity, suggesting the use of a different adjuvant [91].

## 5. Conclusions

Plant-made vaccines are an alternative to control ticks of veterinary importance. The evidence of more than three decades dedicated to optimizing plant-made vaccine platforms and those examples that reached commercialization indicate the feasibility of this technology that has been applied to produce vaccines against parasitic diseases such as fasciolosis, schistosomiasis, coccidiosis, and cysticercosis. Several antigens have demonstrated high protective efficacy in controlling tick infestations in cattle, dogs, and animal models. These antigens are the ideal candidates to be produced in plants and tested in immunological trials against ticks.

## Figures and Tables

**Table 3:** Prototypes of recombinant vaccines against *Haemaphysalis* sp. infection.

Arthropod Specie	Antigen Vaccine Prototype/ Expressión Platform	Pathogen	Animal Model and Immunization Schedule	Main Findings	Reference
*Haemaphysalis longicornis*	Gut protein Acid phosphatase (HL-3) expressed in *Escherichia coli*	N/A	Rabbits: One subcutaneous dose of 0.8 mg of rHL-3 protein emulsified with Freund’s complete adjuvant, with two boosters of 0.8 mg of rHL-3 protein with Freund’s incomplete adjuvant, at 14-day intervals.	Adult female ticks fed on vaccinated rabbits had a 10.6% reduction in engorgement weight and a mortality rate of 28%, compared to those ticks fed on unvaccinated rabbits.	[64]
*Haemaphysalis longicornis*	Salivary protein Longistatin, expressed in *Escherichia coli*	N/A	Mice: Two intramuscular doses of 30 μg of recombinant longistatin emulsified with TiterMax^®^ Gold adjuvant (Sigma), 14 days apart.	-Elicited a specific IgG humoral response. Tick engorgement reduction by 54%, post-engorgement body weight by >11%, and nymphal molt by approximately 34%, with vaccine effectiveness of 73%.	[65]
*Haemaphysalis longicornis*	Paramyosin expressed in *Escherichia coli*	N/A	Rabbits: Three doses of 500 μg recombinant vaccine, the first one adjuvanted with Freund’s complete and two doses with Freund’s incomplete, at 2-week intervals.	-Elicited a specific IgG humoral response. -Reduction in tick engorgement weight, oviposition, and hatchability.	[66]
*Haemaphysalis longicornis*	Lipocalin homologue from *H. longicornis* (HlLIP) expressed in *Escherichia coli*	N/A	Rabbits: Three subcutaneous doses of 500 µg of rHlLIP protein (0.5 mL), the first with complete Freund’s adjuvant, and the subsequent two with incomplete Freund’s adjuvant, at two-week intervals.	-Elicited a specific humoral IgG response. -The vaccination efficacy was 60.17% by the reduction in engorged weight, oviposition and egg hatching rate of ticks.	[67]
*Haemaphysalis longicornis*	Triosephosphate isomerase, expressed in *Escherichia coli*	N/A	Rabbits: three groups (9 rabbits/group). In the experimental group, 0.5 mL rHlTIM (1 μg/μL) mixed with equal volumes of Freund’s complete adjuvant was injected at day 0, followed by two injections with 0.5 mL rHlTIM (1 μg/μL) mixed with equal volumes of Freund’s incomplete adjuvant at intervals of two weeks.	-Elicited a specific humoral IgG response. -Vaccine efficacy of 50.9% by the reductions in engorgement weight, oviposition and hatchability of ticks.	[68]

N/A: No Apply.

**Table 4:** Prototypes of recombinant vaccines against *Amblyomma* spp. infection.

Arthropod Specie	Antigen vaccine Prototype/ Expressión Platform	Pathogen	Animal Model and Immunization Schedule	Main Findings	Reference
*Amblyomma americanum*	The gut proteins: Putative threonyl-tRNA synthetase (2C9), 60S ribosomal proteins L13a (2D10) and L13e (2B7), subolesin and interphase cytoplasm foci protein 45 (2G7), expressed in *Escherichia coli*	N/A	Cattle: 3 subcutaneous doses (weeks 0, 4 and 6) with 100 μg of purified recombinant proteins emulsified with the adjuvant Montanide ISA 50V.	-Elicited a specific IgG humoral response. -An overall efficacy of 30% was obtained with respect to the effect of the vaccine in nymphs and adults, with greater control efficacy for adult ticks, 55%, after immunization with recombinant 2G7 or subolesin.	[69]
*Amblyomma sculptum*	The salivary proteins AsKunitz, AsBasicTail and As8.9kDa, expressed in *Escherichia coli*	N/A	Mice: Three subcutaneous doses of 5 µg of each recombinant protein plus 0.1 mg aluminum hydroxide gel as adjuvant, at 2-week intervals.	-Elicited a specific humoral IgG response. -Vaccine efficacy against *A. sculptum* females was 59.4% with rAsBasicTail and 85% with immunization with rAsKunitz and rAs8.9kDa. The mortality of nymphs fed with immunized mice reached 70–100%.	[70]

**Table 5:** Prototypes of recombinant vaccines against *Hyalomma* spp. infection.

Arthropod Specie	Antigen Vaccine Prototype/ Expressión Platform	Pathogen	Animal Model and Immunization Schedule	Main Findings	Reference
*Hyalomma asiaticum* and *H. anatolicum*	Cathepsin L-like cysteine protease	N/A	Rabbits: Animals were immunized with rHasCPL. Prior to immunization, 200 µg of rHasCPL (0.2 mL) was mixed with equal volume of Inject Alum adjuvant and injected into each rabbit. All rabbits were immunized 3 × at 14-day intervals.	-Vaccine efficacy of 54.8%.	[71]

N/A: No Apply.

**Table 6:** Different antigens that have been tested in immunization trials against cattle ticks in Latin American countries.

Antigen	Efficacy	References
Bm86 (*R. microplus* Bm86 antigen)	Reduction of 70–90% of *Rhipicephalus microplus*	[41]
Bm95 (*R. microplus* Bm95 antigen)	Reduction of 58 and 89% in South American strains of *R. microplus*	[16]
Bm95-msp1a (*R. microplus* Bm95 antigen fused to the *Anaplasma marginale* major surface protein 1a)	64% overall efficacy against *R. microplus*	[55]
RmAQP1 (*R. microplus* aquaporine)	75% and 68% efficacy against Brazilian strains of *R. microplus*	[58]
Ba86 (*Boophilus annulatus* Bm86 ortholog protein)	Efficacy of 83% and 71.5% against Mercedes and Media Joya strains of *R. annulatus* and *R. microplus,* respectively	[43]
Subolesin (*R. microplus*)	51% overall efficacy against *R. microplus*	[20]
Subolesin-mps1a (*R. microplus* Subolesin fused to the *Anaplasma marginale* major surface protein 1a)	60% overall efficacy against *R. microplus*	[55]
Ferritin 2 from: IrFER2 (*I. ricinus*) and RmFER2 (*R. microplus*)	Efficacy of 64% and 72% against *R. microplus* and *R. annulatus,* respectively	[57]
VDAC (*R. microplus*) mitochondrial protein	82% efficacy against *R. microplus*	[49]
P0 (synthetic ribosomal peptide from ticks) conjugated to hemocyanin from *Megathura crenulate*)	Overall efficacy of 90% against *R. sanguineus* feed on rabbits and 96% against a Brazilian strain of *R. microplus*	[61]
Subolesin peptide (from *R. microplus*)	67% efficacy against *R. microplus*	[21]
Bm86 polypeptide (from *R. microplus* Bm86 antigen)	58% efficacy against *R. microplus*	[40]
Chitinase peptide (from *R. microplus*)	71% efficacy	[59]

## Data Availability

Not applicable.
